# Electroacupuncture to improve post-stroke cognitive function and modulate cerebral iron deposition: a randomized controlled trial protocol using MRI

**DOI:** 10.3389/fneur.2025.1708739

**Published:** 2026-01-05

**Authors:** Ning Sun, Fang Xie, Zhe-Tao Wang, Yi-Wei Liu, Hui-Lin Yang, Lei Chen, Jing-Kang Lu, Yi He, Cheng-Qi He, Sha-Xin Liu

**Affiliations:** 1 Rehabilitation Medicine Center and Institute of Rehabilitation Medicine, West China Hospital, Sichuan University, Chengdu, China; 2 Key Laboratory of Rehabilitation Medicine in Sichuan Province, West China Hospital, Sichuan University, Chengdu, China; 3 Acupuncture and Tuina School, Chengdu University of Traditional Chinese Medicine, Chengdu, China; 4 Department of Radiology, West China Hospital, Sichuan University, Chengdu, China

**Keywords:** acupuncture, brain iron deposition, cognitive impairment after stroke, study protocol, MRI

## Abstract

**Background:**

Post-stroke cognitive impairment (PSCI) is common and hampers rehabilitation. Dysregulated iron homeostasis and ferroptosis are implicated in PSCI, yet targeted treatments are lacking. Acupuncture may improve cognition, but its early-intervention efficacy and iron-related mechanisms remain unclear.

**Methods and analysis:**

This single-blind randomized controlled trial will enroll 72 patients with ischemic stroke-related cognitive impairment (IS-CI). Participants will be randomized 1:1 to electroacupuncture (EA) or sham electroacupuncture (sEA) in addition to standard pharmacotherapy and rehabilitation. Participants in the EA group will receive verum stimulation at Baihui (GV20), Sishencong (EX-HN1), Shenting (GV24), and bilateral Neiguan (PC6), along with five adjunct points—Hegu (LI4), Zusanli (ST36), Xuanzhong (GB39), Sanyinjiao (SP6), and Taichong (LR3)—on the affected side. The sEA group will receive sham acupuncture at non-acupoint, non-meridian locations situated 1–2 cun away from the true acupoints. Interventions are delivered 5 times weekly for 4 weeks (20 sessions, 30 min each). The primary outcome is the change in Montreal Cognitive Assessment (MoCA) score from baseline to week 4. Secondary outcomes include Mini-Mental State Examination (MMSE), Modified Barthel Index (MBI), Hamilton Anxiety (HAMA) and Depression (HAMD) scales, Pittsburgh Sleep Quality Index (PSQI), and PSCI incidence at 3-month post-stroke (MoCA <26). A prespecified MRI substudy (*n* = 30) will use 3.0 T quantitative susceptibility mapping (QSM) to quantify regional cerebral iron and explore associations between changes in susceptibility and cognitive measures. Participants and outcome assessors are blinded; allocation is concealed; analyses will follow prespecified statistical plans.

**Conclusion:**

This trial is designed to evaluate the effects of EA on cognitive function in patients with IS-CI and to explore whether changes in cognition are accompanied by changes in cerebral iron deposition measured by QSM. It aims to assess the feasibility and preliminary effects of this intervention and to explore iron-related mechanisms in PSCI using an imaging approach.

**Clinical trial registration:**

http://itmctr.ccebtcm.org.cn/, ITMCTR2025001739.

## Introduction

Stroke is a major acute cerebrovascular disease classified into the ischemic and hemorrhagic subtypes. Ischemic strokes constitute approximately 87% of all cases (1). Globally, it remains the second-leading cause of death and third-leading contributor to disability-adjusted life-years (DALYs) (2), while ranking first in mortality and disability in China (3). Cognitive deficits are common after stroke, and a Chinese expert consensus notes that many patients develop post-stroke cognitive impairment (PSCI) by 3-month post-stroke (4). PSCI primarily affects multiple cognitive domains, including executive function, memory, calculation, visuospatial ability, and language (5), with a global prevalence of approximately 44% and a higher prevalence of about 53.1% among Chinese stroke survivors (6, 7). Cognitive deficits are associated with greater disability, dependence, and higher morbidity, placing a substantial burden on patients, caregivers, and healthcare systems (8). Accordingly, early cognitive assessment and timely intervention are considered critical to improving post-stroke rehabilitation outcomes (4).

The pathogenesis of PSCI is complex and multifactorial, involving direct injury to cognition-related brain regions, disruption of structural and functional connectivity, global neural dysfunction due to acute inflammation and neurotoxicity, and reduced overall brain reserve capacity (8). Recent studies have highlighted the role of dysregulated iron homeostasis in PSCI, particularly implicating ferroptosis, an iron-dependent form of programmed cell death, as a major mechanism of post-ischemic neuronal injury (9). Excess iron can promote oxidative stress and ferroptotic cell death, thereby exacerbating neuronal loss and cognitive decline. Reducing cerebral iron accumulation has therefore been proposed as a potential therapeutic strategy for PSCI (9, 10).

In light of the limited treatment options for PSCI, there is growing interest in non-pharmacological interventions such as acupuncture. Acupuncture is one of the oldest and most widely practiced therapies in Traditional Chinese Medicine (TCM) and is endorsed by the World Health Organization (WHO) (11). Recent systematic reviews and meta-analyses have demonstrated that acupuncture can significantly improve cognitive function among stroke survivors (12–14). However, the neurobiological mechanisms underlying these cognitive benefits remain only partially understood. Preclinical studies using electroacupuncture (EA) in rodent models of ischemic stroke suggest that EA exerts neuroprotective effects by modulating ferroptosis-related pathways. Reported mechanisms include restoring iron homeostasis, inhibiting ferritinophagy, activating the nuclear factor erythroid 2-related factor 2 (Nrf2) antioxidant pathway, and upregulating glutathione peroxidase 4 (GPX4) (15–17). Because EA delivers standardized electrical stimulation at defined frequencies and intensities, it may more reliably engage these iron- and redox-related signaling pathways than manual acupuncture in experimental models. Emerging clinical data suggest that acupuncture can modulate the systemic iron status. In a randomized controlled trial (RCT), adjunctive acupuncture enhanced the response to oral iron, producing larger increases in serum iron, transferrin saturation, and hemoglobin, together with marked reductions in leptin and hepcidin compared with sham acupuncture (18). In another study, needling acupoints led to significantly greater improvements in hemoglobin and red blood cell indices than non-acupoint needling (19). Although these trials assess peripheral rather than cerebral iron, they support the translational plausibility that EA can influence iron-related pathophysiology and provide a clinical rationale to investigate its effects on brain iron deposition in PSCI. Nonetheless, direct evidence that acupuncture alters cerebral iron deposition in stroke survivors is still lacking, and no RCT has yet examined the impact of acupuncture on brain iron load using quantitative neuroimaging techniques.

Therefore, to address these gaps, we have designed an RCT that will utilize 3.0 T magnetic resonance imaging (MRI) with quantitative susceptibility mapping (QSM) to precisely quantify cerebral iron load in patients with ischemic stroke-related cognitive impairment (IS-CI). By combining this neuroimaging approach with detailed cognitive assessments, the trial will comprehensively evaluate both the clinical efficacy of EA and its underlying mechanisms in IS-CI. The specific objectives are:

1. To evaluate the efficacy of EA in improving cognitive function in IS-CI patients;
2. To explore whether EA-related changes in cognition are accompanied by changes in regional cerebral iron deposition quantified by QSM. This imaging approach is intended to provide hypothesis-generating evidence on iron-related mechanisms in PSCI.

## Methods

### Research design

**Figure 1:**
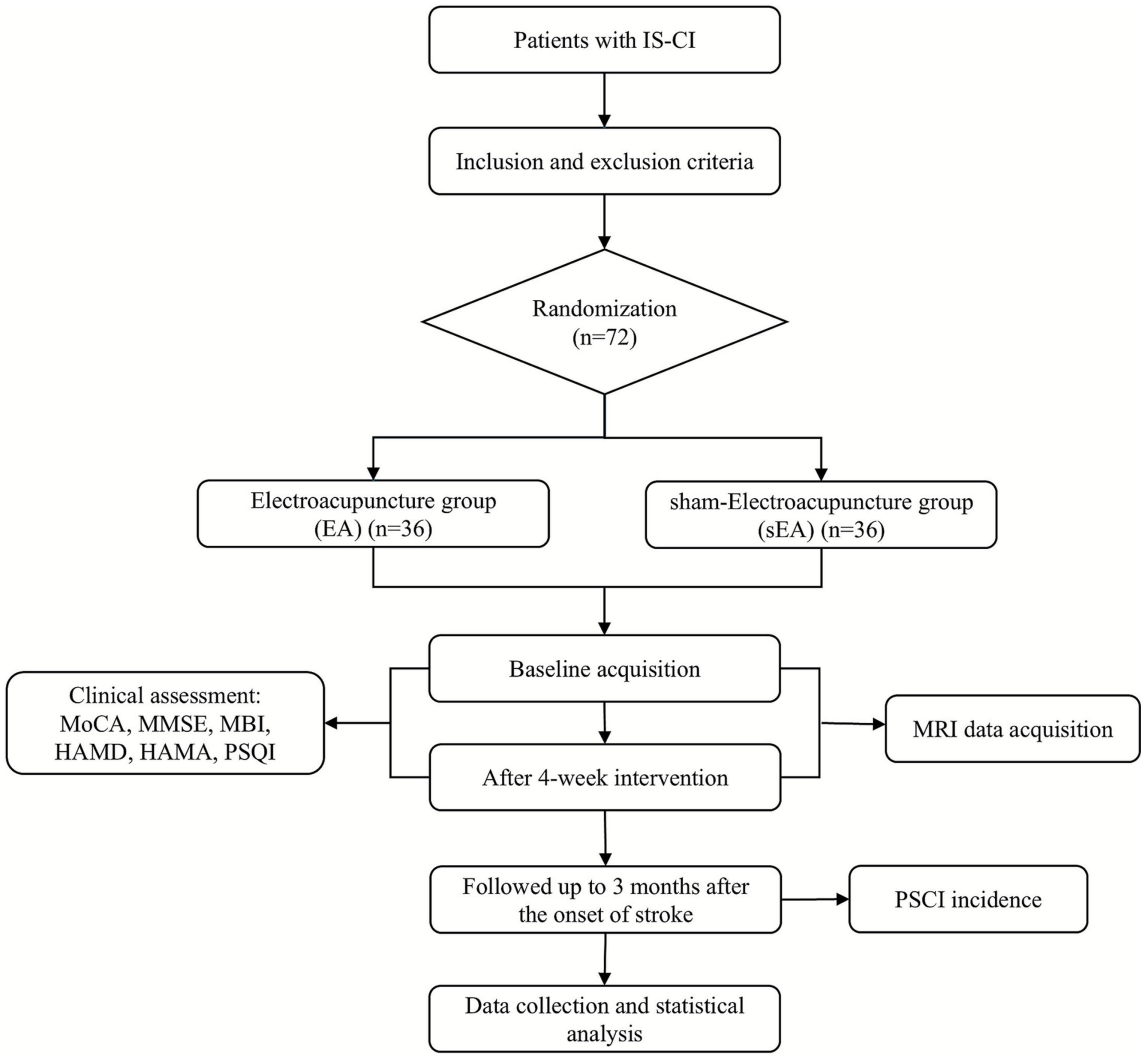
Trial flow chart. IS-CI, ischemic stroke-related cognitive impairment; MoCA, Montreal Cognitive Assessment; MMSE, Mini-Mental State Examination; MBI, Modified Barthel Index; HAMD, Hamilton Depression Rating Scale; HAMA, Hamilton Anxiety Rating Scale; PSQI, Pittsburgh Sleep Quality Index; PSCI, post-stroke cognitive impairment.

This single-blind, RCT will be conducted in the Department of Rehabilitation Medicine, West China Hospital of Sichuan University (Chengdu, China). A total of 72 inpatients with IS-CI will be randomized 1:1 to receive EA or sham electroacupuncture (sEA). Figure 1 outlines the study flow, and Table 1 details the schedule of enrolment, interventions, and assessments.

**Table 1:** Details of the planned visit schedule.

	Study period					
	Baseline (*n* = 72)	Treatment (*n* = 72)				Follow-up (*n* = 72)
Timepoint	0	Week 1	Week 2	Week 3	Week 4	3 Months after onset
Enrollment						
Eligibility screen	×					
Informed consent	×					
Demographic characteristics	×					
Medical history	×					
MRI	×					
Interventions						
Electroacupuncture group		×	×	×	×	
Sham-electroacupuncture group		×	×	×	×	
Assessments						
MoCA	×				×	×
MMSE	×				×	
MBI	×				×	
HAMA	×				×	
HAMD	×				×	
PQSI	×				×	
PSCI incidence						×
Patient safety						
Adverse events		×	×	×	×	

The study received ethics approval (no. 2025-1209) from West China Hospital of Sichuan University on 7 July 2025 and was registered on the International Traditional Medicine Clinical Trial Registry Platform (ITMCTR; registration number: ITMCTR2025001739) on 15 September 2025.

### Diagnostic criteria

Ischemic stroke will be diagnosed according to the 2019 American Heart Association/American Stroke Association (AHA/ASA) Guidelines (20) and the Chinese Guidelines for Acute Ischemic Stroke (2023) (21). Cognitive impairment will be established with reference to the 2023 AHA/ASA scientific statement *“*Cognitive Impairment After Ischemic and Hemorrhagic Stroke*”* (5) as a Montreal Cognitive Assessment (MoCA) score below 26.

#### Inclusion criteria

Inclusion criteria include:

1. Meets both stroke and cognitive impairment definitions;
2. Aged 18–75 years, either of sex;
3. Ischemic stroke onset 7–30 days before enrolment;
4. MoCA < 26 and able to cooperate with acupuncture and assessments;
5. Stable vital signs;
6. National Institutes of Health Stroke Scale (NIHSS) score 1–15;
7. Written informed consent from the patient or legal guardian.

#### Exclusion criteria

Exclusion criteria include:

1. Unstable vital signs or uncontrolled severe cardiopulmonary disease;
2. Previous stroke;
3. Pre-existing cognitive impairment, depression, or other psychiatric disorders;
4. Malignancy, significant hematological disorder, respiratory failure, or active infection;
5. Severe dysfunction of other organs or critical systemic diseases;
6. Severe visual/hearing impairments or aphasia precluding cooperation;
7. Imaging subgroup only: MRI contraindications (metallic implants/electronic devices) or left-handedness.

#### Withdrawal criteria

A participant will be withdrawn from the trial if any of the following conditions occur:

1. Post-enrolment identification of protocol ineligibility.
2. Poor adherence that prevents compliance with the study schedule or procedures.
3. Receipt of another investigational treatment within the previous 3 months.
4. Emergence of serious adverse events or complications requiring discontinuation of the intervention.
5. Voluntary withdrawal for personal, family, or social reasons that preclude completion of the treatment course.

### Sample size

This exploratory trial was primarily designed to estimate effect sizes and assess protocol feasibility. Consistent with pilot-trial recommendations for acupuncture and traditional Chinese medicine (22, 23), we set the target sample size at 30 participants per arm. Allowing for an anticipated 20% attrition, we therefore planned to enroll 36 participants in each group (total *n* = 72). For the MRI substudy, we will randomly select 15 participants per arm (*n* = 30) from the clinical cohorts, in line with Desmond and Glover’s guidance on imaging sample sizes (24). Under a two-tailed *α* = 0.05, this total sample provides approximately 80% power to detect medium-to-large correlations (r ≥ 0.50) between QSM-derived susceptibility measures and cognitive outcomes, based on Fisher’s z transformation in G*Power 3.1. Smaller associations will be interpreted with caution, and the MRI substudy is therefore primarily intended to provide preliminary estimates of effect sizes and variance components to guide the design of future, adequately powered imaging trials. With 15 participants per arm, the substudy will also have approximately 80% power to detect between-group differences in 4-week changes in regional susceptibility of approximately 0.8 standard deviations (Cohen’s d), whereas smaller treatment effects on QSM will be treated as exploratory and described descriptively.

### Randomization and blinding

Participants will be randomly assigned to either the EA group or the sEA group using a random number table. The randomization sequence will be prepared in advance by an independent investigator who is not involved in participant enrolment, treatment, or outcome assessment. Allocation will be concealed using sequentially numbered, opaque, and tamper-evident envelopes. After confirming eligibility and completing baseline assessments, the treating acupuncturist will open the next envelope in sequence to determine group allocation and initiate the assigned intervention.

Due to the nature of the intervention, blinding treatment providers is not feasible. Therefore, a single-blind design will be used. Participants and outcome assessors will remain blinded to group allocation, while acupuncturists will be aware of treatment assignment but restricted to intervention delivery only. Data entry and statistical analysis will be performed by an independent third party to minimize bias.

To assess the credibility of blinding, we will adopt a procedure similar to that used in recent acupuncture RCTs (25). At the end of the 4-week intervention, participants will be asked to indicate which treatment they believe they have received (EA, sEA, or uncertain). The distribution of responses will be summarized descriptively to evaluate the success of blinding. Based on the Patients’ Expectancy Scale of Acupuncture (PESA) (26), we will include a brief expectancy questionnaire at baseline, asking participants to rate, on a 5-point Likert scale, how much they believe acupuncture will help their post-stroke cognitive recovery.

### Intervention and grouping

Participants will be randomly assigned to either the EA group or the sEA group. All participants will continue standard pharmacotherapy and rehabilitation during the study. Each participant will receive 20 treatment sessions (5 sessions per week for 4 weeks), each lasting 30 min.

#### EA group

**Figure 2:**
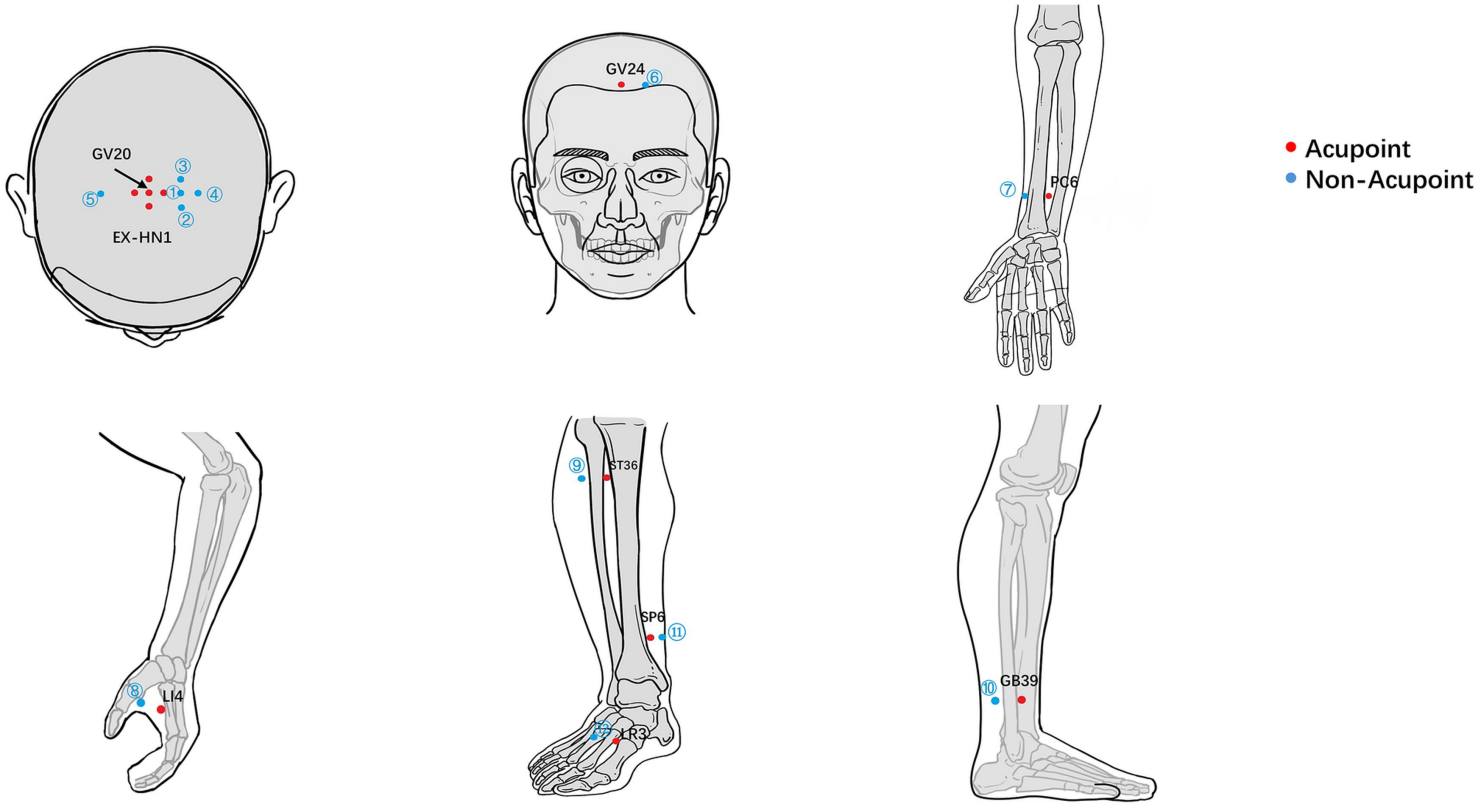
Location of acupoints and non-acupoints.

Participants in the EA group will receive verum stimulation at Baihui (GV20), Sishencong (EX-HN1), Shenting (GV24), and bilateral Neiguan (PC6), along with five adjunct points—Hegu (LI4), Zusanli (ST36), Xuanzhong (GB39), Sanyinjiao (SP6), and Taichong (LR3)—on the affected side. Three adjunct points will be selected per session based on clinical presentation. Acupoint locations will follow the WHO Standard Acupuncture Point Locations (see Figure 2; Supplementary Table S1).

After skin disinfection with 75% ethanol, sterile disposable stainless-steel filiform needles (0.25 mm × 40 mm) will be inserted. For scalp points (GV20, EX-HN1, GV24), needles will be inserted obliquely at a 15° angle to the scalp to reach the subgaleal layer, then advanced without rotation to a depth of 0.8 cun. For body points, insertion will follow standard depth and angle: PC6, LI4, ST36, and SP6 will be needled perpendicularly to a depth of 1.0 cun; GB39 perpendicularly to 0.8 cun; and LR3 obliquely to 0.8 cun. A balanced reinforcing–reducing technique will be applied to elicit the deqi sensation. EA will be delivered using the Hua Tuo SDZ-III device with four electrode pairs (GV20–GV24, left–right EX-HN1, and bilateral PC6 with auxiliary needles). Alternating sparse-dense waveforms (2 Hz/10 Hz) will be applied, with stimulation intensity set at 1–2 mA depending on patient tolerance. Identical procedures and device cues were maintained across groups to preserve blinding, while the absence of electrical output in the sEA group ensured inert stimulation.

#### sEA group

Participants in the sEA group will receive sham acupuncture at non-acupoint, non-meridian locations situated 1–2 cun away from the true acupoints. Locations will follow the SHARE guidelines (27) and the relevant systematic review (28), with locations specified in Figure 2 and Supplementary Table S2. Needles (0.25 mm × 25 mm) will be inserted subcutaneously (scalp) or perpendicularly (body) to 0.3 cun, without manipulation or deqi. Electrodes will be placed in the same configuration as in the EA group. To maintain participant blinding, the Hua Tuo SDZ-III device will appear fully operational, with power indicators and auditory signals activated. However, the internal wiring of the electrode leads will be disconnected to ensure that no electrical current is delivered to the non-acupoints.

By using non-meridian, non-acupoint locations and superficial, non-manipulated needling, we aimed to minimize acupoint-specific effects while maintaining credibility, in line with current recommendations for sham acupuncture controls (27, 28). Nonetheless, we acknowledge that even superficial needle insertion and cutaneous stimulation may elicit non-specific sensory or segmental neuromodulatory effects, so any between-group differences are likely to represent conservative estimates of the specific effects of EA.

All treatments will be administered by physicians and therapists, who will receive protocol-specific training. Before trial initiation, they will attend a structured workshop based on a written treatment manual detailing acupoint location, needling depth and angle, manipulation, stimulation parameters, and session duration for both EA and sEA. Adherence to the protocol will be monitored via treatment logs and periodic supervision to ensure consistency across practitioners.

### Safety evaluation

Safety monitoring will focus on adverse events (AEs) related to EA, including vasovagal reactions (dizziness, nausea, pallor, transient hypotension or syncope), intolerable needling pain, local hematoma or bruising, bleeding, infection or abscess at needle sites, needle breakage, and other post-needling reactions. All AEs will be recorded on standardized case report forms, noting the onset of time, duration, severity, relationship to the intervention, actions taken, and outcome. Serious adverse events (SAEs) will be defined according to Good Clinical Practice. Participants with repeated vasovagal episodes, severe AEs, or any SAE judged related to the intervention will discontinue study treatment and may be withdrawn if further participation is unsafe. All SAEs will be reported to the principal investigator and the institutional ethics committee within 24 h and will be managed according to the Data and Safety Monitoring procedures of West China Hospital.

### Outcome measures

#### Primary outcome measurement

The primary outcome is the change in MoCA scores from baseline to the end of the 4-week treatment period. This time point corresponds to completion of the EA course and the early subacute rehabilitation phase, when treatment intensity and neuroplastic potential are highest and short-term clinical decision-making is often made. MoCA is a validated screening tool for global cognitive function, covering seven domains: visuospatial/executive function, naming, attention, language, abstraction, delayed recall, and orientation (total score = 30). Using the standardized cutoff of <26 established for cognitive impairment detection, lower scores denote greater severity of cognitive dysfunction (29). To address longer-term clinical relevance, MoCA will also be reassessed at 3 months after stroke onset, and PSCI incidence at 3 months will be analyzed as a key secondary outcome.

#### Secondary outcomes

##### Cognitive function

The Mini-Mental State Examination (MMSE) will be used to assess global cognition. This 30-item scale evaluates seven domains: temporal orientation, spatial orientation, immediate recall, attention and calculation, delayed recall, language, and visuospatial abilities. Education-adjusted cutoff scores for cognitive impairment are: ≤17 for illiterate individuals, ≤20 for primary school, and ≤24 for junior secondary education or above (30).

##### Activities of daily living

Functional independence will be measured using the Modified Barthel Index (MBI), which assesses 10 daily activities (e.g., feeding, bathing, and mobility). Total scores range from 0 to 100, with lower scores indicating greater dependency (31).

##### Anxiety

Anxiety will be assessed using the Hamilton Anxiety Rating Scale (HAMA-14), which comprises 14 items divided equally into somatic anxiety and psychic anxiety. A total score ≥7 indicates probable clinical anxiety, while <7 falls within the normal range, with higher scores reflecting greater severity (32).

##### Depression

Depressive symptoms were evaluated via the 17-item Hamilton Depression Rating Scale (HAMD-17), covering seven domains: anxiety/somatization, weight changes, cognitive impairment, diurnal variation, psychomotor retardation, sleep disturbances, and hopelessness. Scores ≥7 suggest possible depression (<7 = normal), where elevated scores denote increased depressive severity (33).

##### Sleep quality

The Pittsburgh Sleep Quality Index (PSQI) will be used to evaluate subjective sleep quality over the past month. The index includes seven components scored from 0 to 3, producing a global score ranging from 0 to 21. Higher scores reflect poorer sleep quality (34).

##### PSCI incidence

In accordance with the 2023 AHA/ASA scientific statement (5), PSCI will be defined as a MoCA score <26 at the 3-month post-stroke evaluation. PSCI incidence will be calculated as the number of confirmed cases/total number of enrolled participants.

#### Assessment timepoints

The primary outcome (MoCA) and most secondary outcomes (MMSE, MBI, HAMA, HAMD, and PSQI) will be assessed twice: at baseline and at the end of the 4-week treatment period. PSCI incidence, defined based on MoCA <26, will be assessed once at 3 months after stroke onset.

### MRI data acquisition

All MRI examinations will be performed at the MRI Center of West China Hospital, Sichuan University, on a 3.0 T scanner (Discovery MR750; GE Healthcare, Milwaukee, USA) equipped with an eight-channel phased-array head coil. Before each examination, participants will be instructed to minimize head motion, and their heads will be stabilized with foam pads. Scans will be acquired with the participant lying supine and remaining alert throughout. After acquisition, two trained neuroimaging analysts will visually inspect the magnitude and phase images for artifacts, including motion, signal dropout, and severe susceptibility distortions. Datasets with pronounced motion artifacts that preclude reliable phase unwrapping will be repeated where feasible; if repetition is not possible, the scan will be excluded from QSM analyses, and the reason will be documented.

A high-resolution structural dataset will be obtained with a three-dimensional T1-weighted spoiled gradient-echo sequence (TR/TE = 6.2/2.5 ms, field of view = 256 × 256 mm^2^, data matrix = 256 × 256, voxel size = 1.0 × 1.0 × 1.0 mm^3^). QSM will be derived from a gradient-recalled echo sequence (flip angle = 17°, field of view = 229 × 229 mm^2^, voxel size = 0.7 × 0.7 × 4.0 mm^3^, number of slices = 52, slice thickness = 4 mm).

### Clinical data analysis

Statistical analyses will be performed using IBM SPSS 26.0 (IBM, Armonk, NY, USA). The primary efficacy analyses will follow the intention-to-treat (ITT) principle (35), including all randomized participants analyzed according to their allocated group, irrespective of treatment adherence. A per-protocol (PP) population will be defined as participants who complete at least 16 of the 20 planned treatment sessions (≥80% adherence) and have the primary endpoint (week-4 MoCA) assessed. For the MRI substudy, the PP set will include participants with analyzable QSM data at both baseline and week 4.

MoCA change from baseline to week 4 will be the sole primary endpoint, and hypothesis testing for this outcome will use a two-sided significance level of 0.05. Prespecified secondary clinical outcomes (MMSE, MBI, HAMA, HAMD, PSQI, and PSCI incidence) will be analyzed without formal adjustment for multiplicity and will be interpreted as exploratory. For these endpoints, we will report effect sizes with 95% confidence intervals and regard *p*-values as descriptive, in order to avoid over-interpretation of nominal statistical significance in this pilot trial. Multiplicity for imaging analyses across QSM regions of interest will be controlled using false-discovery-rate correction as described below.

For the primary endpoint (change in MoCA from baseline to week 4), if missing data at week 4 exceeds 5% of randomized participants, multiple imputation under a missing-at-random assumption will be used as the main ITT analysis (36, 37). For secondary outcomes, analyses will primarily use available cases, and sensitivity analyses with multiple imputation may be conducted if missingness is substantial. Reasons for missing data will be documented wherever possible.

Continuous variables will be presented as mean ± standard deviation (SD) when normally distributed or median with an interquartile range (IQR) for non-normally distributed data. Between-group comparisons will utilize independent samples t-tests for normally distributed variables and Mann–Whitney U-tests otherwise. Within-group pre-post comparisons will use the Wilcoxon signed-rank test. Correlation analyses will preferentially apply Pearson’s method; if either variable violates normality, Spearman’s rank correlation will be substituted. To control for confounders, covariates including age, sex, education level, stroke severity (baseline NIHSS score), and time from stroke onset to enrolment will be incorporated into multivariable linear regression models to evaluate between-group differences in the primary endpoint and key secondary cognitive outcomes. Where available, additional vascular and imaging-related factors, such as stroke subtype, lesion side, and vascular territory, lesion volume, baseline white matter hyperintensity burden (e.g., Fazekas score), receipt of intravenous thrombolysis, and major vascular comorbidities, will be explored as candidate covariates in sensitivity analyses.

### Neuroimaging data analysis

We will process the GRE data to generate QSM maps using the standard pipeline implemented in the SuscEptibility mapping PIpeline tool for phase (SEPIA) images toolbox run under MATLAB (MathWorks, Natick, MA), following established methodologies (38). Whole-brain magnitude images will be skull-stripped using the BET tool from the FMRIB Software Library, integrated within the MEDI toolbox. The overall procedure consisted of three main steps: (1) Total field recovery will be achieved through echo phase combination via the optimum weights method (39), followed by phase unwrapping using SEGUE (40). (2) Background field removal will then be performed on the unwrapped phase images by applying the Laplacian boundary value approach (41). (3) Finally, quantitative susceptibility maps will be reconstructed using the STAR-QSM algorithm for dipole inversion (42).

Region-of-interest (ROI) definition and extraction will follow an *a priori*, region-based approach. We will focus on deep grey matter and limbic structures that are susceptible to post-stroke iron accumulation and implicated in cognition, including the bilateral caudate nucleus, putamen, globus pallidus, thalamus, hippocampus, red nucleus, substantia nigra, and dentate nucleus, informed by previous QSM studies of brain iron and clinical outcomes (43–46). For each participant, the T1-weighted structural image will be co-registered to the QSM map, and subcortical ROIs will be segmented using a standard atlas-based method implemented in FSL. The resulting ROI masks will be resampled into QSM space and visually inspected. Mean susceptibility values (ppm) within each ROI will be extracted at baseline and at week 4. All registrations and ROI segmentations will be visually checked by two independent neuroimaging analysts. Any discrepancies will be resolved by consensus, and if necessary, the processing pipeline will be refined before final extraction. All image preprocessing and QSM analyses will be performed on de-identified datasets that are coded by study ID, and the analysts will remain blinded to treatment allocation and clinical outcome data until the primary analyses have been completed.

Statistical analysis will be performed in SPSS 26.0 (IBM, Armonk, NY, USA). Within-group changes in susceptibility (post–pre) are tested with paired-samples *t*-tests, and between-group differences are assessed with independent-samples t-tests. Pearson correlation coefficients will be calculated to examine associations between changes in ROI-based susceptibility and corresponding changes in cognitive, functional, emotional, and sleep measures; if normality is violated for either variable, Spearman’s rank correlation will be used. Data normality will be assessed with the Shapiro–Wilk test. Two-sided *p*-values of < 0.05 will be considered statistically significant, and false-discovery-rate correction will be applied across the predefined ROIs to control for type I error. Given the 4-week interval and modest imaging sample size, the QSM substudy is not powered to detect small absolute susceptibility changes. We will therefore focus on within-subject changes in ROI-based susceptibility and on medium-to-large associations with cognitive outcomes, interpreting all imaging findings as exploratory.

## Discussion

PSCI compromises post-stroke functional recovery and quality of life, placing substantial burdens on patients and caregivers. Early intervention in the early subacute phase may help mitigate subsequent cognitive decline. Recent systematic reviews and meta-analyses have suggested that acupuncture may confer modest benefits on stroke-related outcomes and complications, while also highlighting heterogeneity in trial quality and a paucity of mechanistic imaging studies (47). Against this background, the present RCT is designed to evaluate whether EA could improve cognitive performance in patients with IS-CI and to explore whether any cognitive benefits are accompanied by changes in cerebral iron deposition measured by QSM. If successful, this study could provide a mechanism-informed, non-pharmacological strategy for improving post-stroke cognitive outcomes.

According to TCM, PSCI is categorized as “post-stroke mental dullness,” attributed to blood stasis obstructing the brain’s collateral channels and disrupting the connection between cerebral and visceral qi (48). Governor Vessel acupoints such as Baihui (GV20), Sishencong (EX-HN1), and Shenting (GV24), together with the Pericardium meridian point Neiguan (PC6), are among the most frequently used points for post-stroke cognitive dysfunction (49). Recent randomized trials have shown that EA at GV20 and GV24 can modulate cognition-related brain networks and improve MoCA scores (50, 51), while stimulation of EX-HN1 and PC6 has been associated with altered functional connectivity in default-mode and salience networks and increased low-frequency oscillatory power in cognition-related regions (52, 53). Based on this converging evidence, we selected GV20, EX-HN1, GV24, and bilateral PC6 as the primary acupoints. To preserve blinding, the control group receives sham needle stimulation at adjacent non-meridian, non-acupoint locations with superficial, non-manipulated insertion and no electrical output, which is expected to minimize acupoint-specific effects while maintaining credibility.

Iron is an essential micronutrient in the brain, involved in neurotransmitter synthesis, myelin formation, and mitochondrial energy production (54, 55). However, ischemic stroke can disrupt iron homeostasis, resulting in excess iron accumulation in affected and connected brain regions and triggering iron-dependent oxidative neurotoxicity (ferroptosis) (56, 57). Radiological and histopathological studies have demonstrated progressive iron deposition in structures such as the ipsilateral thalamus, substantia nigra, and basal ganglia after stroke, and higher post-stroke iron burden in deep grey matter has been linked to poorer neurological outcomes (57–59). The degree of post-stroke iron accumulation in these regions correlates with worse neurological outcomes (56, 58, 59). These observations support cerebral iron overload as a clinically relevant, potentially modifiable factor in post-stroke recovery and provide a rationale to investigate brain iron as a mechanistic target in PSCI.

QSM is a gradient-echo based MRI post-processing technique that reconstructs tissue magnetic susceptibility maps from phase images. Because paramagnetic iron is a major source of susceptibility contrast in the brain, higher QSM values closely reflect increased regional iron content and have been widely used to characterize iron accumulation in brain aging, neurodegenerative diseases, and cerebrovascular disorders (44, 60, 61). In patients with acute IS, a recent QSM study reported that higher iron content in deep grey matter nuclei was associated with poorer functional outcome (43), supporting cerebral iron burden as a clinically meaningful imaging marker in this population. These advances indicate that QSM provides a quantitative and relatively specific *in vivo* measure of brain iron load, making it particularly suitable for testing ferroptosis-related hypotheses in PSCI. However, the magnitude and time course of intervention-related changes in QSM after stroke are not yet established in humans. In this trial, QSM outcomes will therefore be treated as exploratory mechanistic markers to provide effect-size estimates and generate hypotheses.

Preclinical studies suggest that acupuncture may confer neuroprotection in ischemic stroke by modulating ferroptosis and related pathways. In rodent models, EA has been reported to improve neurological function and reduce infarct volume, with effects comparable to those of iron chelators, by regulating iron-metabolism-related targets and inhibiting neuronal ferroptosis (15, 62). Other studies have shown that EA activates the Nrf2/SLC7A11/GPX4 antioxidant pathway, suppresses lipid peroxidation, preserves mitochondrial integrity, and attenuates ischemia mitochondrial integrity Nrf2/SLC7A11/GPX4 antioxidant pathway (16, 17). In addition to these anti-ferroptosis effects, acupuncture may inhibit neuronal apoptosis, enhance synaptic plasticity, dampen central and peripheral inflammation, and stabilize cerebral energy metabolism (63–65). Most of this evidence, however, comes from animal or *in vitro* experiments. Our trial does not directly measure molecular markers of ferroptosis; instead, QSM provides a non-invasive, iron-sensitive imaging readout that can generate hypothesis-generating evidence on whether iron-related mechanisms are engaged in human IS-CI patients receiving EA.

### Innovation and limitations

To our knowledge, this is the first randomized trial in patients with IS-CI that integrates EA with 3.0 T QSM to quantitatively assess cerebral iron deposition as a mechanistic biomarker. Unlike previous acupuncture trials that have primarily used functional MRI, diffusion tensor imaging (DTI), or positron emission tomography (PET) to evaluate hemodynamic, connectivity, or metabolic changes, our study focuses on QSM-based iron mapping to directly probe a ferroptosis-related mechanism. However, because no molecular or biochemical markers of ferroptosis are measured, any mechanistic inferences from QSM findings will be indirect and hypothesis-generating. Methodological safeguards include concealed allocation, single-blinding of participants and outcome assessors, separation of acupuncturists, evaluators, and statisticians, and blinded data analysis. Nevertheless, practitioner blinding is not feasible, and the single-blind design carries a risk of performance bias, despite standardized training and protocolized procedures. The sham control used in this trial is also unlikely to be completely physiologically inert, as even superficial needle insertion at non-meridian, non-acupoint locations may induce non-specific sensory or segmental neuromodulatory effects. Consequently, any between-group differences are likely to represent conservative estimates of the specific effects of EA. Finally, the modest sample size and the choice of a 4-week primary endpoint, rather than 3 months, limit the ability to detect small effects and to fully characterize long-term cognitive recovery, although MoCA and PSCI incidence at 3 months are included as key secondary outcomes.

## Conclusion

This protocol describes a single-blind RCT evaluating the effects of EA on cognition in patients with IS-CI. We will also examine whether changes in cognitive performance are accompanied by changes in QSM-derived cerebral iron deposition. The trial is designed to generate prospective randomized data on EA as a potential non-pharmacological option for PSCI and to provide hypothesis-generating imaging evidence on iron-related mechanisms, thereby informing future, larger mechanistic studies.
