# Supplementary material for: Electroacupuncture to improve post-stroke cognitive function and modulate cerebral iron deposition: a randomized controlled trial protocol using MRI
Source: Front Neurol. 2026 Jan 5;16:1708739. doi: 10.3389/fneur.2025.1708739 (PMC12812620; doi:10.3389/fneur.2025.1708739)
Supplement: Supplementary file 1 [file Table_1.docx]

**Table S1. Acupoints used in the verum acupuncture group**

| **Acupoints** | **Anatomical location** |
| --- | --- |
| **Primary** | |
| Baihui (GV20) | On the head, 5 B-cun superior to the anterior hairline, on the anterior median line. |
| Sishencong (EX-HN1) | On the top of the head, 1 B-cun each to the anterior, posterior, left, and right of the Baihui (GV20) acupoint, totaling 4 acupoints. |
| Shenting (GV24) | On the head, 0.5 B-cun superior to the an terior hairline, on the anterior median line. |
| Neiguan (PC6) | On the anterior aspect of the forearm, between the tendons of the palmaris longus and the flexor carpi radialis, 2 B-cun proxi mal to the palmar wrist crease. |
| **Adjunct (affected side)** | |
| Hegu (LI4) | On the dorsum of the hand, radial to the midpoint of the second metacarpal bone. |
| Zusanli (ST36) | On the anterior aspect of the leg, on the line connecting ST35 with ST41, 3 B-cun inferior to ST35. |
| Xuanzhong (GB39) | On the fibular aspect of the leg, anterior to the fibula, 3 B-cun proximal to the prominence of the lateral malleolus. |
| Sanyinjiao (SP6) | On the tibial aspect of the leg, posterior to the medial border of the tibia, 3 B-cun superior to the prominence of the medial malleolus. |
| Taichong (LR3) | On the dorsum of the foot, between the first and second metatarsal bones, in the de pression distal to the junction of the bases of the two bones, over the dorsalis pedis artery. |

**Table S2. Non-acupoints used in the sham acupuncture group**

| **Non-acupoints** | **Anatomical location** |
| --- | --- |
| Non-acupoint 1 | 2 cun left-lateral to the GV20. |
| Non-acupoint 2 | 2 cun left-lateral to the aterior EX-HN1. |
| Non-acupoint 3 | 2 cun left-lateral to the posterior EX-HN1. |
| Non-acupoint 4 | 2 cun left-lateral to the left EX-HN1. |
| Non-acupoint 5 | 2 cun right-lateral to the right EX-HN1. |
| Non-acupoint 6 | 2 cun left-lateral to the GV24. |
| Non-acupoint 7 | 2 cun radial to the PC6. (bilateral) |
| Non-acupoint 8 | 2 cun radial to the LI4. (affected side) |
| Non-acupoint 9 | 2 cun lateral to the ST36. (affected side) |
| Non-acupoint 10 | 2 cun lateral to the GB39. (affected side) |
| Non-acupoint 11 | 2 cun posterior to the SP6. (affected side) |
| Non-acupoint 12 | 2 cun lateral to the LR3.( affected side) |
